# A Conversation with Greta Heydenrych

**DOI:** 10.1021/acscentsci.3c00116

**Published:** 2023-02-03

**Authors:** Dalmeet Singh Chawla

For most chemists, the International Union of Pure and Applied Chemistry (IUPAC), is the organization that gives elements their names and decides what terminology researchers should use in scientific papers.

**Figure:**
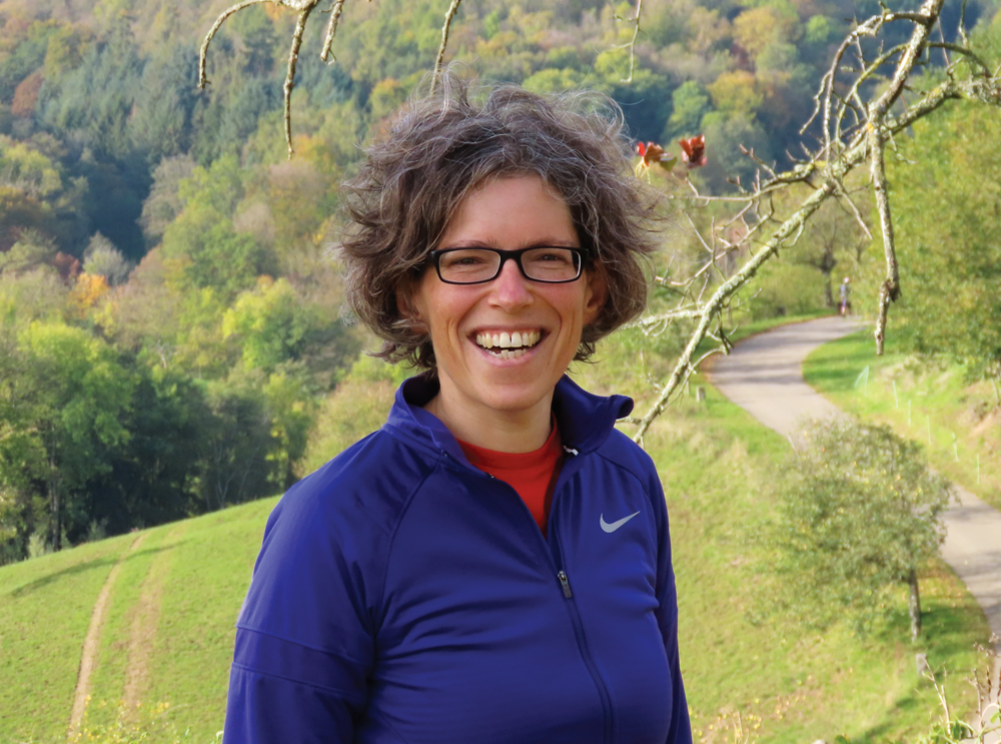
Credit: Volker Jacob

But Greta Heydenrych, who was appointed IUPAC’s executive director in October, can attest that a lot of work goes on behind the scenes to ensure that chemists can communicate and thrive. To her, IUPAC is more than just its *Compendium of Chemical Terminology*, the iconic “Gold Book” of chemical nomenclature. It’s also a group of largely volunteer chemists who want to make it easier to share research and foster the chemistry community through IUPAC’s outreach and education programs.

Dalmeet Singh Chawla spoke with Heydenrych about why chemists should embrace standardization, the direction in which she hopes to lead IUPAC, and the challenges the organization faces. This interview has been edited for length and clarity.

## What exactly does IUPAC do?

Quite a lot. What is most well known is our work on nomenclature, standards, conventions and so on. That’s the core of what we do, but we do much more than that.

We try to do a lot to connect people across the world. IUPAC sponsors or funds projects and task groups each year related to nomenclature, data standards and topics in chemistry education. We have initiatives like the Global Women’s Breakfast—an annual event that runs in dozens of countries under a different theme each year—and we have experimental kits that we are taking into schools that give teachers the ability to train children in critical scientific principles without the need to run a lab. IUPAC is doing a lot to make sure everybody who wants to be part of the chemistry community can be a part of it.

## What’s your ultimate goal becoming the executive director of IUPAC?

I would say the big thing for the next 5–10 years—and even longer, because chemistry is a complex subject—is digital standardization. There’s a huge need for standardization in data reporting and in the data itself: to make sure that people adopt data-sharing practices, to encourage people to develop data-sharing systems in such a way that those tools can talk to each other and to develop them in such a way that open-source versions are available. As a subject discipline, we’re probably somewhat behind the physicists and biologists.

## How does IUPAC introduce new nomenclature or a new standard for data reporting?

It’s a long process. When someone realizes that the community needs some new guidelines or a standard, they would submit a project proposal. That person can come from our subject-focused divisions such as inorganic chemistry or analytical chemistry, our interdisciplinary committees, or from the wider chemistry community.

In their proposal, they say “OK, we need to tidy up this area of chemistry,” and they work with experts in the field to draw up potential guidelines or standards. In doing so, they look, for example, at how naming is done in the community or how molecular structures are encoded to make them machine readable, aiming to reconcile these approaches and trying to come up with a decision that is as consistent as possible.

Those materials then go out for peer review. Typically we ask them to submit and publish in our journal, *Pure and Applied Chemistry*, where it will go through a very, very rigorous review process—in some sense even more stringent than scholarly papers usually do. That’s because we need to be really sure that all the data and all the decisions being made are rock solid and make sense based on whatever other recommendations we have already made.

After the recommendations successfully pass peer review, they are published. After that, we make an announcement on our website, our newsletter, social media, and through our magazine, *Chemistry International*.

## How do you make sure everybody follows your recommendations?

This is a question we ask ourselves as well. It’s tricky, especially because the dissemination of science has become quite fragmented with the rise of preprint servers, social media, and so on.

We encourage people to check one of our eight Color Books, which contain information about our chemical nomenclature, terminology, and symbols. [IUPAC’s Color Books are a series of reference books that are informally named after the color of each book’s cover.—Ed.] We also work closely with publishers to make sure they have information about our recommendations in their journals’ publishing guidelines for authors.

With the digitization of chemistry, the rise of open data, and the ways that people communicate with one another changing so quickly, we have to find new ways to make sure people are aware of our recommendations. We also have to go further to make sure people understand our recommendations and how to implement them.

## Do you think terms can ever be coined and spread organically? Or do they need to be imposed top down?

I think it probably is a bit of both. I don’t like to think of IUPAC as imposing rules. Rather, I think of IUPAC as the global organization that provides all chemists with a common language.

It’s in the interest of all chemists to maintain this common language and adhere to it so we all know what the other is talking about. All these new materials that we make—they need names, and you can’t just call them any old thing.

## What sectors of chemistry are precipitating the most changes?

Based on my previous role as a journal editor, I would say the biggest output had been in materials science, especially as related to energy materials. Supramolecular chemistry, specifically metal–organic frameworks and related compounds, is a fast-moving field as well.

And our work on establishing digital representations of compounds, such as that with the InChI [International Chemical Identifier] Trust, and development of other digital standards, such as IUPAC’s work with the International Science Council’s Committee on Data, will only increase in importance over the next few years.

## What do you want the chemistry community to know about IUPAC?

IUPAC is part of the chemistry community, but the first thing to say would be that the paid staff of IUPAC is tiny—currently, we are only four people! Almost all our work is done by dedicated volunteers who are very, very generous with their time, expertise, and input. Without them, IUPAC would not be able to function. And therefore, we are always on the lookout for anyone from the broader chemistry community who might be willing to contribute to IUPAC’s work.

*Dalmeet Singh Chawla is a freelance contributor to*Chemical & Engineering News*, an independent news publication of the American Chemical Society.*

